# Eigen-analysis reveals components supporting super-resolution imaging of blinking fluorophores

**DOI:** 10.1038/s41598-017-04544-5

**Published:** 2017-06-30

**Authors:** Krishna Agarwal, Dilip K. Prasad

**Affiliations:** 1 BioSystems and Micromechanics Inter-Disciplinary Research Group, Singapore-MIT Alliance for Research and Technology, Singapore, 138602 Singapore; 2 Department of Physics and Technology, UiT-The Arctic University of Norway, 9037 Tromsø, Norway; 3 School of Computer Science and Engineering, Nanyang Technological University, Singapore, 639798 Singapore

## Abstract

This paper presents eigen-analysis of image stack of blinking fluorophores to identify the components that enable super-resolved imaging of blinking fluorophores. Eigen-analysis reveals that the contributions of spatial distribution of fluorophores and their temporal photon emission characteristics can be completely separated. While cross-emitter cross-pixel information of spatial distribution that permits super-resolution is encoded in two matrices, temporal statistics weigh the contribution of these matrices to the measured data. The properties and conditions of exploitation of these matrices are investigated. Con-temporary super-resolution imaging methods that use blinking for super-resolution are studied in the context of the presented analysis. Besides providing insight into the capabilities and limitations of existing super-resolution methods, the analysis shall help in designing better super-resolution techniques that directly exploit these matrices.

## Introduction

An excited fluorophore (referred to as emitter) such as a quantum dot, a molecule of organic dye, or a fluorescent protein, looses energy by radiative mechanism (i.e. emitting a photon of the emission wavelength) or non-radiative mechanisms (such as through heat or vibration), or utilizes other photonic pathways such as induction of reversible dark states^1–3^ photo-activation^1^, photo-switching^2–4^, Förster resonance energy transfer^2, 5^ etc. The occurrence of photon emission and non-radiative relaxation^6–8^, is stochastic. The photonic mechanisms of regulating emissions can be controlled to a large extent by manipulation of photo-chemical environment and utilization of customized dyes^9, 10^. We collectively call the intermittent emissions, stochastic or photochemically regulated, as blinking in the current work.

Blinking is exploited by several state-of-the-art computational super-resolution imaging methods such as super-resolution optical fluctuation imaging (SOFI)^11–13^, entropy-based super-resolution imaging (ESI)^14^, Bayesian analysis of the blinking and bleaching (3B)^15^, stochastic optical reconstruction microscopy (STORM)^16^, photo-activated localization microscopy (PALM)^17, 18^, spatial covariance reconstructive (SCORE) super-resolution fluorescence microscopy^19^, super-resolution radial fluctuations (SRRF)^20^, and multiple signal classification algorithm (MUSICAL)^21^. While imaging, the number of emissions from an emitter during one frame stochastically varies from the next or previous frames due to blinking. The spatial distribution and blinking of the emitters in the focal volume get mapped through the optical point spread function (PSF) of the imaging system and result into an intensity pattern on camera pixels which appears as temporally fluctuating images. Several of these images are recorded and the resulting image stack is used by the computational methods to obtain super-resolved images of the system^22^.

This paper presents eigen-analysis of such image stack of blinking emitters. This analysis establishes the role of blinking statistics and spatial distribution of emitters and identifies the cross-pixel and cross-emitter terms that embed super-resolvability. The eigen-analysis and its results are used as a common framework to investigate the state-of-the-art super-resolution techniques that use blinking.

## Results

### Eigen-analysis

We assume the following for the eigen-analysis:

- All emitters are similar, with the same photon counting statistics for the frame acquisition time *T*. Every emitter’s photon emission distribution has mean value of *μ* and standard deviation of *σ*.
- Each emitter’s photon emission is independent of any other emitter’s behaviour. As an implication, the covariance of the photon emission of any two emitters is zero.
- The number of frames is large enough that the mean and standard deviations of the actual photo emissions from the emitters converge to *μ* and *σ*, respectively. Additionally, the emitters do not photo-bleach or transit into irreversible or extremely dark states during image stack acquisition.
- Imaging system is diffraction limited. The point spread function of the system spans at least a few pixels.

These assumptions are quite practical and implicitly used in most super-resolution imaging methods that exploit blinking. Important mathematical notations used in this paper are listed in Supplementary Table S1. We perform eigen-analysis of the matrix

1 $${\bf{J}}={\bf{I}}{{\bf{I}}}^{{\rm{T}}}$$

where *I* is a matrix of size *N* × *K* containing the image stack of *M* emitters located at ${r}_{m}^{^{\prime} }$, *m* = 1 to *M* at *N* pixels centered at *r*_*n*_, *n* = 1 to *N* in *K* frames (see supplementary information S1 on imaging model) and the superscript T is the transpose operator. Using assumptions A1–A3, **J** may be written as (see supplementary information S2 for derivation from eq. (1) to eq. (2)):

2 $${\bf{J}}=K{\bf{GO}}{{\bf{G}}}^{{\rm{T}}}$$

where

3 $${\bf{O}}=({\sigma }^{2}{ {\mathcal I} }_{\{M\}}+{\mu }^{2}{ {\mathcal L} }_{\{M\times M\}})$$

and **G** is a matrix of size *N* × *M* and its *m*th column ${\bar{G}}_{m}$ column represents the image of an emitter. ${\bar{G}}_{m}$ is related to the optical PSF (as shown in supplementary information S1). ${\mathcal I}$ and ${\mathcal L}$ denote an identity matrix and an all-ones matrix, respectively. Their dimensions are specified in their subscript.

**O** is a symmetric circulant matrix with all diagonal elements equal to *σ* ^2^ + *μ* ^2^ and all non-diagonal elements equal to *μ* ^2^. Eigen-analysis of **O**, given in supplementary information S3, reveals that eigenvectors of **O** can be interpret as Fourier transform operators^23^. Thus, eigen-decomposition of **J** comprises of spatial frequencies of **G**. Upon substitution of eigen-decomposition of **O** in eq. (2) and further algebraic manipulation (details in supplementary information S4), we obtain:

4 $${\bf{J}}={c}_{1}{{\bf{C}}}_{1}+{c}_{2}({{\bf{C}}}_{2}-{{\bf{C}}}_{3})$$

where

5 $${c}_{1}=KM({\sigma }^{2}+M{\mu }^{2});\quad {c}_{2}=K(M-\mathrm{1)}{\sigma }^{2}$$

6 $${{\bf{C}}}_{1}=\frac{1}{{M}^{2}}\bar{\tilde{G}}{\bar{\tilde{G}}}^{{\rm{T}}}$$

7 $${{\bf{C}}}_{2}=\frac{1}{M}\sum _{m=1}^{M}\,{\bar{G}}_{m}{\bar{G}}_{m}^{{\rm{T}}}$$

8 $${{\bf{C}}}_{3}=\frac{1}{M(M-\mathrm{1)}}\sum _{m=1}^{M}\,\sum _{m^{\prime} =1;m^{\prime} \ne m}^{M}\,{\bar{G}}_{{m}^{^{\prime} }}{\bar{G}}_{m}^{{\rm{T}}}$$

and $\bar{\tilde{G}}={\sum }_{m}\,{\bar{G}}_{m}$. $\bar{\tilde{G}}$ is proportional to the mean image of the image stack as discussed in supplementary information S1. An extension to the case of non-independent emitters (assumption A2 relaxed) is given in supplementary information S5 and we note that only the coefficients *c*_1_ and *c*_2_ change in this situation.

The above result provides the insight that the spatial distribution of the pixels and the temporal distribution of blinking can be separated from each other. While the matrices **C**_{1,2,3}_ contain information pertaining the spatial distribution only of the emitters, the temporal blinking characteristics weigh the contribution of these matrices and thus modulate the content pertaining super-resolution in the measured image stack.

### Spatial distribution and the roles of C_{1,2,3}_ in super-resolution

In the following, we analyse the implications of **C**_{1,2,3}_ on super-resolution. We refer to 〈*f*(*n*, *m*)〉_*m*_ as ‘raw moment over emitters’ for verbal simplicity instead of the more rigorous ‘raw moment of the bivariate function *f*(*n*, *m*) over the variable *m*. Similarly, we refer to 〈*f*(*n*, *m*)〉_*n*_ as ‘raw moment over pixels’. Further, we refer to *f*(*n*, *m*) *f*(*n*′, *m*) as ‘cross-pixel’ term and *f*(*n*, *m*) *f*(*n*, *m*′) as ‘cross-emitter’ term. The elements **C**_{1,2,3}_ (*n*′, *n*) of matrices **C**_{1,2,3}_ comprise of the following raw moments:

9 $${C}_{1}(n^{\prime} ,n)={\langle G(n^{\prime} ,m)\rangle }_{m}{\langle G(n,m)\rangle }_{m}$$

10 $${C}_{2}(n^{\prime} ,n)={\langle G(n^{\prime} ,m)G(n,m)\rangle }_{m}$$

11 $${C}_{3}(n^{\prime} ,n)={\langle G(n^{\prime} ,m^{\prime} )G(n,m)\rangle }_{m,m^{\prime} \ne m}$$

Thus, **C**_1_ corresponds to cross-pixel product of first order raw moment over emitters, **C**_2_ corresponds to second order cross-pixel raw moment over emitters, and **C**_3_ corresponds to second order cross-pixel cross-emitter raw moment over emitters.

**Figure 1:**
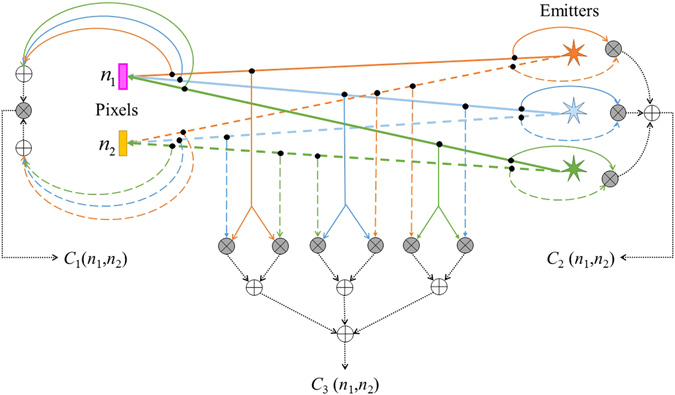
Illustration of the elements of matrices **C**_{1,2,3}_ is given here. Note the order of products (gray circles with cross sign) and sums (white circles with plus sign).

Specifically, **C**_1_ corresponds to cross-pixel product of mean image of image stack (as also noted in Fig. 1). It essentially emulates the effect of all emitters emitting the same number of photons simultaneously. Thus, **C**_1_ does not support super-resolution. However, the diagonal elements of **C**_1_ (i.e. *n* = *n*′) give auto-correlation of the mean image with itself and through this, **C**_1_ supports contrast enhancement.

**C**_2_ corresponds to the raw moment over emitters of cross-pixel product of image of each emitter individually. As shown in Fig. 1, the cross-pixel terms in **C**_2_ are computed before the averaging over emitters is performed. Computationally, it makes the effective PSF as squared of the optical PSF, and thus $\sqrt{2}$ times sharper than the optical PSF. Through this effect, it contributes towards resolution better than the optical resolution. It essentially supports super-resolution through autocorrelation terms with respect to the emitters.

**C**_3_ contains cross-pixel cross-emitter terms, which are then averaged over all emitter pairs (*m*, *m*′); $m\ne m^{\prime}$. Thus, in comparison to **C**_2_, this term sharpens the PSF with respect to not only the pixels, but also the emitters. Thus, it supports super-resolution through cross-correlation terms with respect to the emitters. Also note that

12 $$M{C}_{1}(n^{\prime} ,n)={C}_{2}(n^{\prime} ,n)+(M-\mathrm{1)}{C}_{3}(n^{\prime} ,n)$$

While **C**_2_ has higher spatial frequencies than **C**_1_ due to narrower PSF, **C**_3_ suppresses the high frequency component from **C**_2_ to yield **C**_1_. Thus, **C**_3_ has a high frequency component antisymmetric to **C**_2_. Lastly, while a certain additive combination of **C**_2_ and **C**_3_ suppresses high frequency component as noted in eq. (12), the subtraction **C**_2_ − **C**_3_ suppresses the lowest frequency component.

### Examples illustrating C_{1,2,3}_

We further illustrate the roles of **C**_{1,2,3}_ using two synthetic 1-dimensional examples. Simulation details are provided in supplementary information S6) and it suffices to say that the full width at half maximum (FWHM) of the imaging system is 187 nm and the resolution limit according to Rayleigh criterion^24^ is 222 nm. Since the matrices are independent of emission kinetics, simulation of the properties of matrices does not require simulation of emission kinetics.

**Figure 2:**
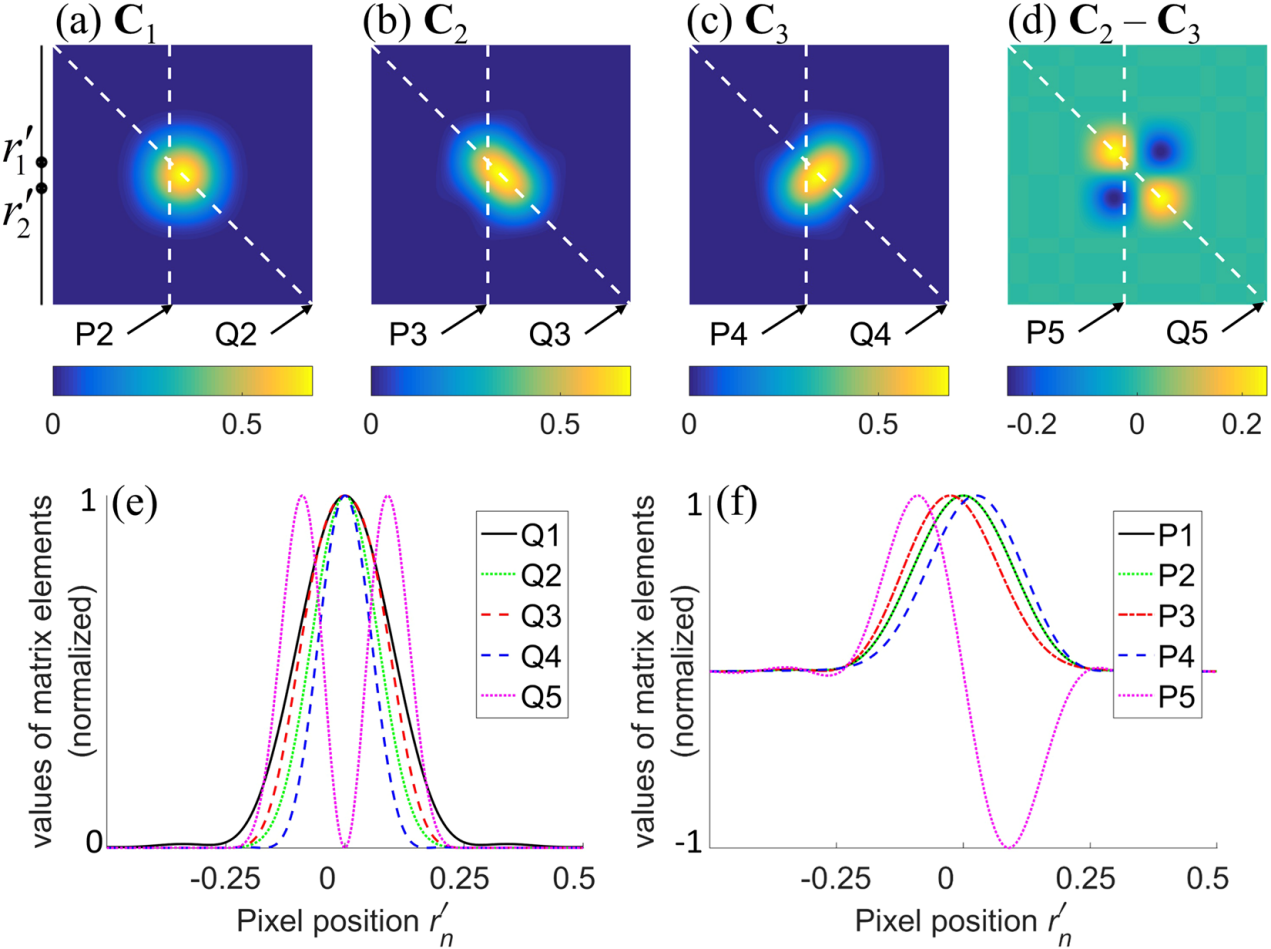
Matrices **C**_1_, **C**_2_, **C**_3_, (**C**_2_ − **C**_3_) for example 1 are shown in (**a**–**d**). The distance between the emitters is 100 nm. The plots for *n*′ = *n* are shown in (**e**) and the plots for ${r}_{n}^{^{\prime} }=-50$ nm are shown in (**f**). Plots Q1 and P1 in (**e**,**f**) are both equal to $\bar{\tilde{G}}$. All the plots in (**e**,**f**) are normalized by their maximum amplitudes.

In the first example (example 1), an ideal one-dimensional camera with infinitesimally small pixel size is considered. The emitters are separated by distance Δ*y* = 100 nm. The pixel array is along the *y*–axis and symmetric to the emitters. The matrices **C**_1_, **C**_2_, **C**_3_, and **C**_2_ − **C**_3_ are shown in Fig. 2(a–d) respectively. It is seen that **C**_2_ and **C**_3_ indicate the presence of more than one emitters; **C**_3_ is antisymmetric, and; **C**_2_(*n*, *n*) − **C**_3_(*n*, *n*) is small at pixels between the emitters.

**Figure 3:**
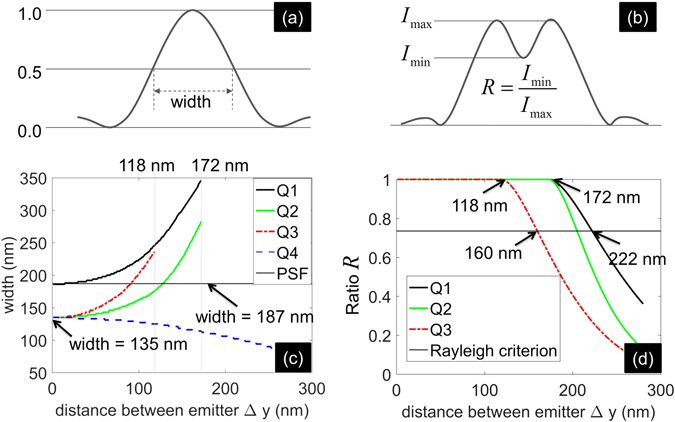
Properties of the matrices **C**_{1,2,3}_ are illustrated through FWHM (definition in (**a**), result in (**c**)) and minima to maxima ratio (defined in (**b**), result in (**d**)) of Q1–Q4 as functions of Δ*y*.

Figure 2(e) plots the mean image $\bar{\tilde{G}}$ (Q1) and the diagonal elements (*n* = *n*′) of matrices **C**_1_, **C**_2_, **C**_3_, and **C**_2_ − **C**_3_. It is seen that the plot of **C**_3_ is the sharpest, followed by **C**_1_, **C**_2_, and $\bar{\tilde{G}}$, respectively. Figure 3(c) shows their FWHM as a function of Δ*y*. It confirms that the FWHM of Q2 (**C**_1_) is smaller than that of Q3 (**C**_2_) when Δ*y* is so small (<118 nm) that neither **C**_1_ nor **C**_2_ can resolve the emitters (i.e. Q2 and Q3 have only one maximum each). This indicates better sharpening of image through **C**_1_ but not the resolvability of the emitters.

The width corresponding to **C**_3_ (Q4) is smaller than both Q2 and Q3, thus corroborating sharpening due to both cross-emitter and cross-pixel terms. As the distance between emitters become large, the cross-emitter components attenuate and Q4 becomes even sharper. In the limiting case, Δ*y*→0, the widths (135 nm) of Q2–Q4 are all $\sqrt{2}$ times the width of the PSF and the width of the mean image (both equal to 187 nm).

Sharpening of PSF in **C**_2_ is illustrated through minima to maxima intensity ratio *R* defined in Fig. 3(b) and plotted in Fig. 3(d) as a function of Δ*y*. Here, it becomes obvious that **C**_1_ merely enhances the contrast of the mean image $\bar{\tilde{G}}$ of emitters that are already resolvable by the optical system whereas **C**_2_ actually improves the resolution by a factor of $\sqrt{2}$.

Lastly, we note that **C**_2_ − **C**_3_ incorporates higher spatial frequencies than **C**_{1,2,3}_ and information about resolvability of emitters, as noted in both the Q5 and P5 plots in Fig. 2(e,d) in comparison to Q1–Q4 and P1–P4 plots.

**Figure 4:**
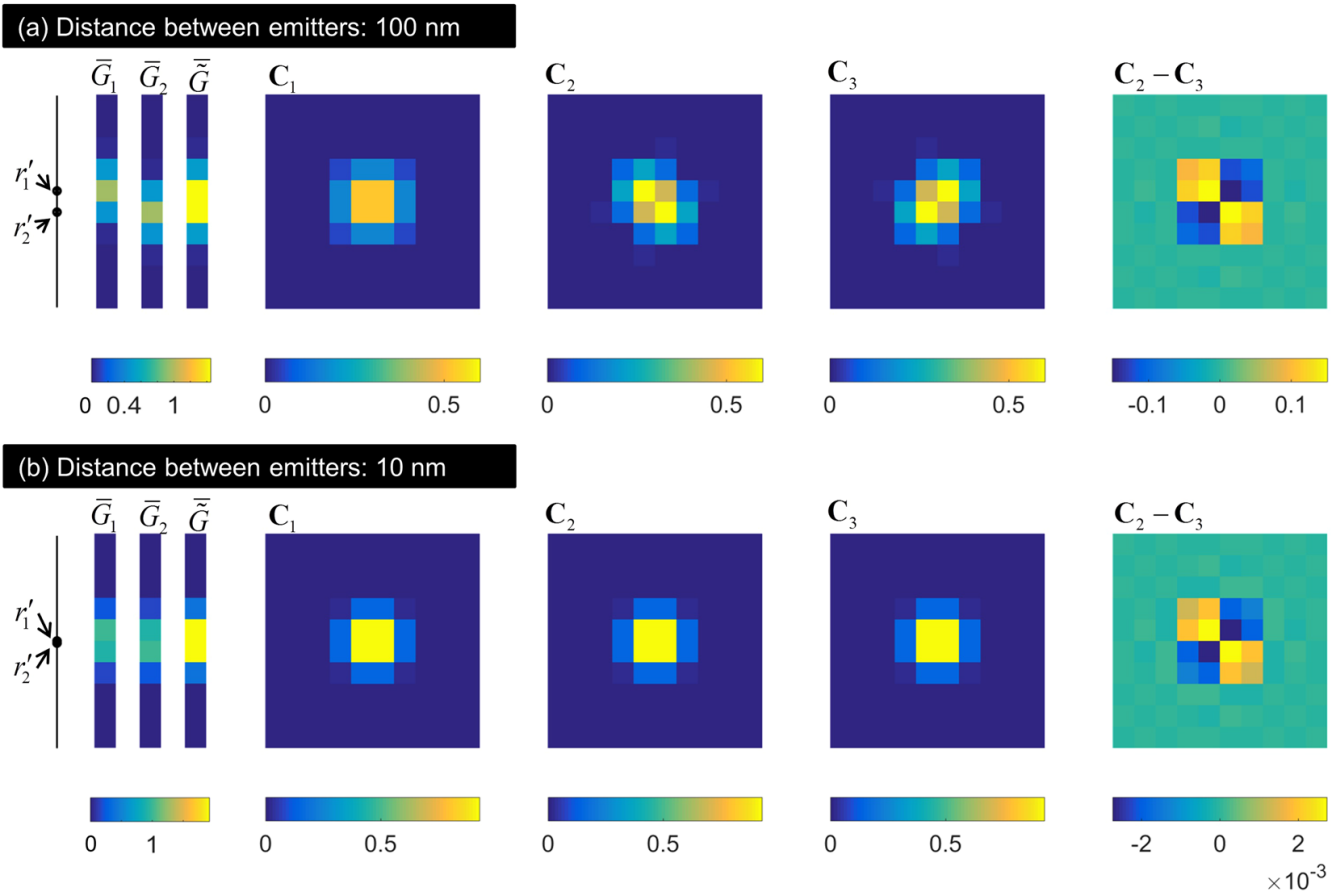
The effect of finite pixel size on the matrices **C**_1_, **C**_2_, **C**_3_, (**C**_2_ − **C**_3_) is demonstrated here through Example 2. (**a**,**b**) correspond to distance between emitters being 100 nm and 10 nm, respectively. The pixel size is 100 nm.

In the second example (example 2), we include the effect of practical pixel size. The image is taken along the *y*–axis using 10 pixels, each of size 100 nm, placed symmetric to the emitters. We consider two values of Δ*y* and show ${\bar{G}}_{1}$, ${\bar{G}}_{2}$, $\bar{\tilde{G}}$, **C**_1_, **C**_2_, **C**_3_, and **C**_2_ − **C**_3_ in Fig. 4. For Δ*y* = 100 nm (Fig. 4(a)), the nature of the matrices in unaffected except discretization due to finite pixel size. Even for Δ*y* = 10 nm (Fig. 4(b)), the nature of **C**_2_ − **C**_3_ is the same although its maximum value (referred to as magnitude of matrix for simplicity) is significantly smaller than the magnitudes of **C**_1_, **C**_2_, **C**_3_, as seen in Fig. 4(b). This implies that its contribution may be negligible in the presence of noise.

**Figure 5:**
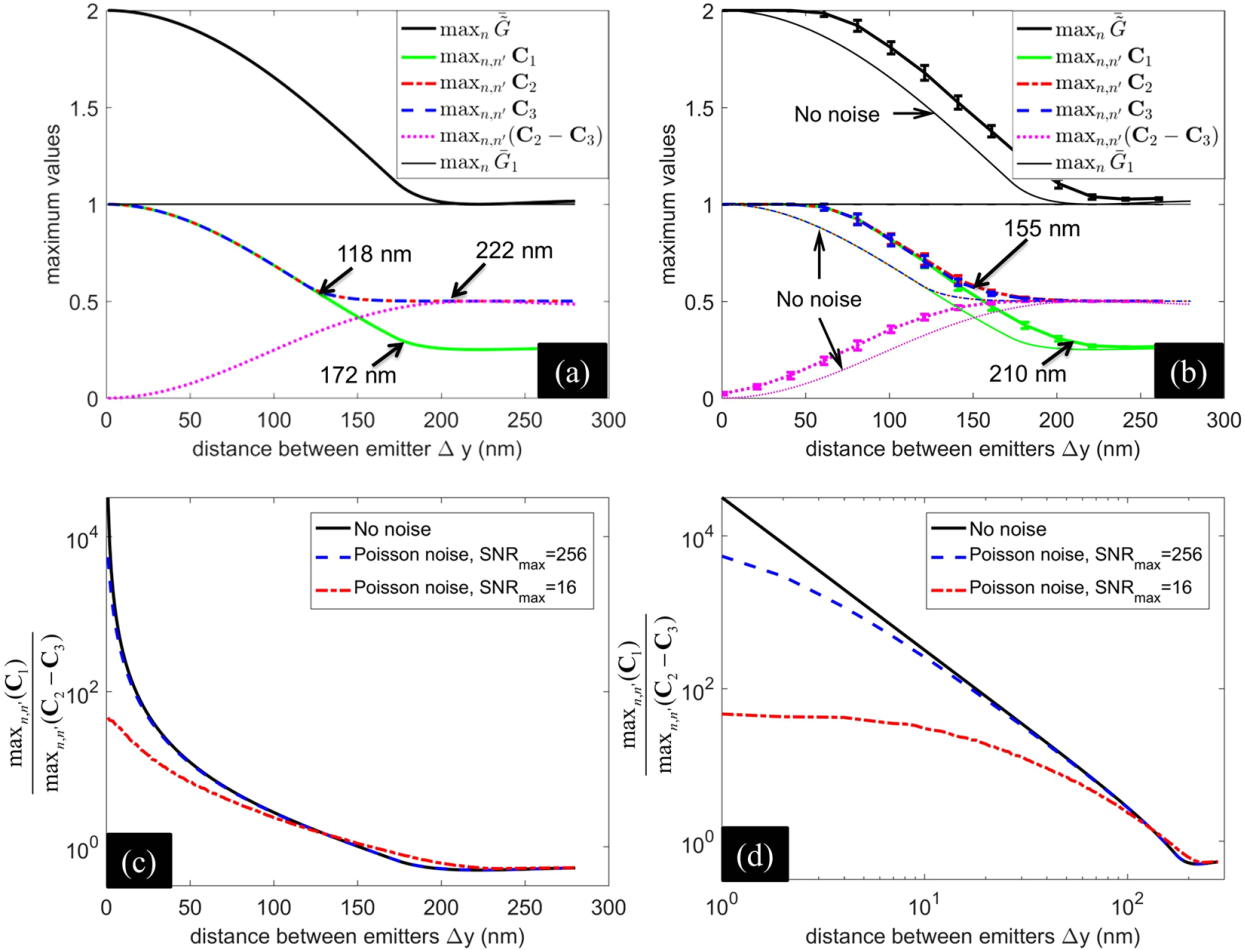
Comparison of magnitudes (maximum values) of matrices **C**_1_, **C**_2_, **C**_3_, and **C**_2_ − **C**_3_ is presented here. (**a**) Provides a direct comparison of magnitudes, assuming no measurement noise. (**b**) Compares their magnitudes for Poisson noise with maximum SNR 16. (**c**) Compares the ratio of the magnitude of **C**_1_ to the magnitude of **C**_2_ − **C**_3_. (**d**) is the same as (**c**) but with both horizontal and vertical axes in logarithmic scale.

We examine the magnitudes of these matrices as a function of Δ*y* in Fig. 5(a). Finite pixel size of 100 nm is used. Beyond the resolution limit of 222 nm, **C**_2_, **C**_3_, and (**C**_2_ − **C**_3_) converge to 0.5 whereas **C**_1_ converges to 0.25. This is neither surprising nor interesting since the emitters are resolved anyway. The point Δ*y* = 118 nm is interesting, since the minima to maxima intensity ratio *R* for Q3 (corresponding to **C**_2_) falls below 1 at Δ*y* = 118 in Fig. 3(d), indicating the minimum Δ*y* such that **C**_2_ can resolve the emitters. In Fig. 5(a), **C**_1_, **C**_2_, and **C**_3_ have the same magnitudes up to Δ*y* = 118 nm, after which the magnitude of **C**_1_ is smaller than the magnitudes of **C**_2_ and **C**_3_. **C**_2_ and **C**_3_ have an inflection point at Δ*y* = 118 nm. The inflection point of **C**_1_ and $\bar{\tilde{G}}$ occurs at Δ*y* = 172 nm which corresponds to the fall of the value of *R* for Q2 and Q1 below 1 in Fig. 3(d).

Since, **C**_2_ − **C**_3_ incorporates all the content pertaining super-resolution that cannot be captured by the optical system without blinking (see eq. (4)), we plot the ratio of the magnitude of **C**_1_ to the magnitude of **C**_2_ − **C**_3_ in Fig. 5(c). It indicates that typically the magnitude of **C**_1_ is orders of magnitude larger than **C**_2_ − **C**_3_. Thus, direct exploitation of **C**_2_ − **C**_3_ is difficult in the presence of noise. Super-resolution techniques that use either **C**_2_ or **C**_3_ individually or linear combination *a* **C**_2_ + *b* **C**_3_ such that *a*, *b* are real numbers and *b*/*a* is not equal to −1 or *M* − 1 (see eq. (12)) can support super-resolution despite the presence of noise. We observed an interesting characteristic reported in Fig. 5(d) that the logarithm of ratio of the magnitude of **C**_1_ to the magnitude of **C**_2_ − **C**_3_ decreases linearly with the logarithm of Δ*y*. The observation may provide additional insight in the future.

In order to consider the effect of noise on the comparative magnitudes of matrices **C**_{1,2,3}_, we include Poisson noise with maximum signal-to-noise ratio (SNR) equal to 16 to the matrix **G** containing the images of individual emitters. We use this noisy matrix **G** to compute the matrices **C**_{1,2,3}_. We perform this operation for hundred independent executions of noise. The mean and standard deviations corresponding to the plots in Fig. 5(a) are plotted in Fig. 5(b). It is seen that noise has significant effect on the magnitudes of the matrices. The inflection points in the case of SNR = 16 are shifted by about 40 nm. We include the effect of noise in Fig. 5(c,d) as well. It is interesting to note that noise reduces the ratio of the magnitude of **C**_1_ to the magnitude of **C**_2_ − **C**_3_. This is because **C**_2_ − **C**_3_ retains the high frequency component characterizing the noise. This is evident in the increase in the magnitude of **C**_2_ − **C**_3_ in the presence of noise, as noted in Fig. 5(b). Thus, although the ratio is improved, **C**_2_ − **C**_3_ is not conducive to super-resolution because of the noise retained. Further, the effect of noise in degrading resolution is not negligible due to the shift in the inflexion points of **C**_2_ and **C**_3_ as noted in Fig. 5(b).

### Examples of super-resolution supported by C_2_ and C_3_

**Figure 6:**
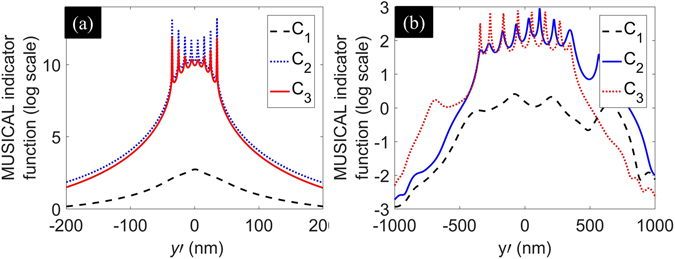
MUSICAL images using only **C**_1_, **C**_2_ or **C**_3_ for one-dimensional example of 8 emitters separated by 10 nm (**a**) and 100 nm (**b**). (**b**) Corresponds to Poisson noise with SNR 20. It is evident that **C**_1_ is incapable of supporting resolution whereas **C**_2_ and **C**_3_ can support super-resolution, subject to noise.

Here, we show the ability of **C**_2_ and **C**_3_ and the inability of **C**_1_ to support super-resolution. Similar to the previous example, we consider 1-dimensional sample and image regions. Image region has 40 pixels of 100 nm each. Sample has 8 emitters placed symmetrically along the *y*-axis at a separation of 10 nm between them. The other details are the same as the previous examples. We compute the one-dimensional eigenimages of **C**_1_ and apply MUSICAL^21^ on them. In order to study only the effect of the matrix, we do not use sliding window and the soft window function. This is equivalent to a single sliding window and Gaussian soft window function of variance equals to infinity. Further, we use the non-linear power factor of MUSICAL *α* = 1, so that *α* does not introduce additional non-linearity. Similar to **C**_1_, we compute MUSICAL images using **C**_2_ and **C**_3_ as well. The results are shown in Fig. 6(a). It is seen that MUSICAL image of **C**_1_ cannot resolve any emitter, whereas MUSICAL images of both **C**_2_ and **C**_3_ are able to resolve all the emitters correctly.

We also include an example of eight emitters placed at a distance of 100 nm. Poisson noise with peak SNR 20 is used to create noisy matrix **G**, which is then used for computing the matrices **C**_{1,2,3}_. The results are shown in Fig. 6(b). It is seen that MUSICAL image of **C**_1_ cannot resolve any emitter. Eight peaks are detected using both **C**_2_ and **C**_3_. Additional peaks at approximately *y*′ = 700 nm are noted for the MUSICAL curves corresponding to **C**_1_ and **C**_2_, which are due to noise. A peak at *y*′ = −700 nm is observed in MUSICAL curve for **C**_3_, which corresponds to the antisymmetry of **C**_3_.

### Blinking statistics and the role of *c*_1_ and *c*_2_ in super-resolution

The ratio of *c*_1_ and *c*_2_ determine the ratio mean image (through **C**_1_) to the content pertaining super-resolution (through **C**_2_ and **C**_3_). It is notable from Equation (5) that *c*_1_, *c*_2_ > 0 and *c*_2_/*c*_1_ < 1 in any situation, even if a single emitter is present. This implies that the mean image always forms the major component in the image stack. Nevertheless, the higher the ratio *c*_2_/*c*_1_, the better is the scope for super-resolution. The ratio *c*_2_/*c*_1_ is characterized by the photon-counting statistics (which determines *μ* and *σ*) and the density of emitters through *M*. Smaller values of *μ* ^2^/*σ* ^2^ and *M* are desirable for larger value of *c*_2_/*c*_1_.

**Figure 7:**
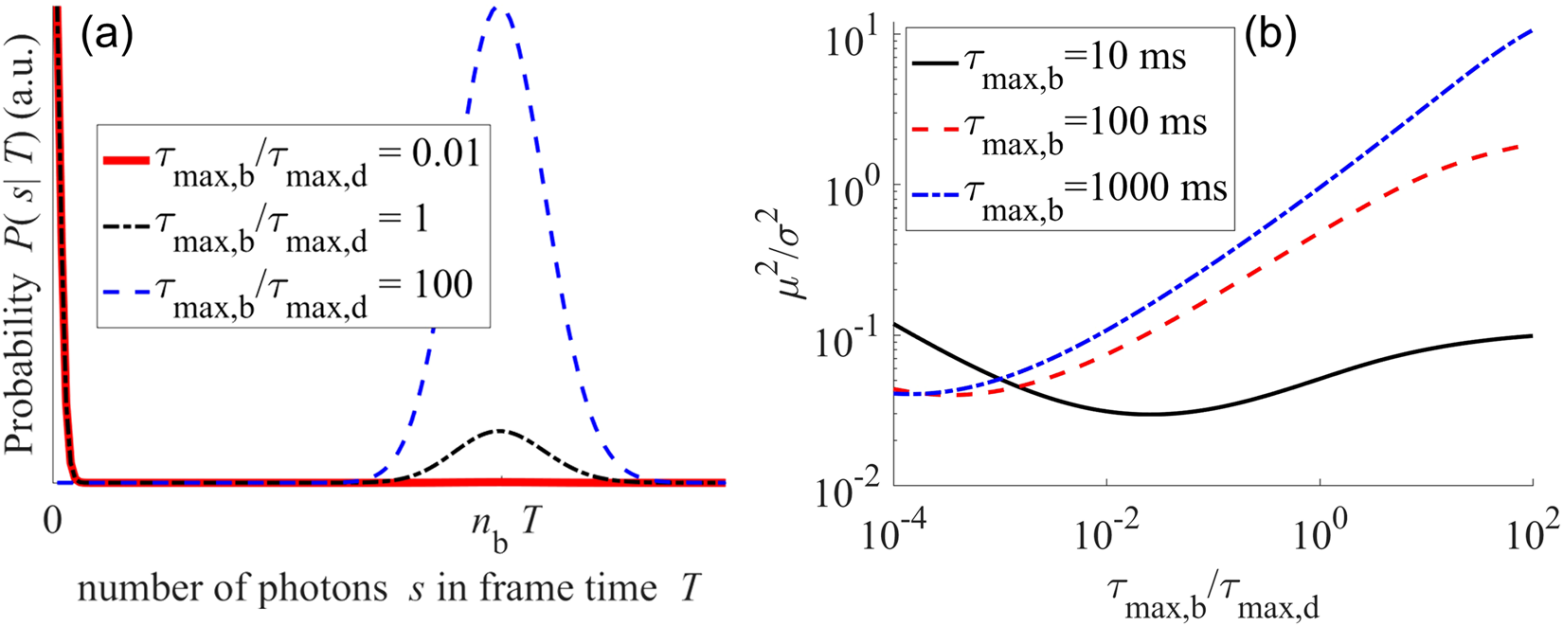
Variety of photon counting statistics are shown in (**a**). For frame acquisition time *T* = 10 ms, the ratio *μ* ^2^/*σ* ^2^ is plotted as a function of ${\tau }_{\max ,{b}}/{\tau }_{\max ,{d}}$ in (**b**). Additional result is given in supplementary information S8.

First, we consider the ratio *μ* ^2^/*σ* ^2^. We use the blinking kinetics of quantum dots as an example for this purpose. The modeling and simulation of photon counting statistics of quantum dots^8^ are given in the supplementary information S7. Characteristic photon-count distributions for three forms of photon-counting statistics due to the maximum time scales of dark states (${\tau }_{\max ,d}$) relative to maximum time scales of bright states (${\tau }_{\max ,b}$) are given in Fig. 7(a). Value of ${\tau }_{\max ,b}/{\tau }_{\max ,d}$ less than 1 indicates longer dark states than bright states and vice versa. Thus, the ratio ${\tau }_{\max ,b}/{\tau }_{\max ,d}$ indicates the temporal sparsity of blinking and translates to spatial sparsity of emissions if the frame acquisition time $T$ is sufficiently low.

Further, the effect of the time scales of blinking with respect to $T$ is illustrated in Fig. 7(b). The condition $T\sim {\tau }_{\max ,b}$ implies that blinking occurs at time scales comparable to the frame acquisition time. $T\ll {\tau }_{\max ,b}$ implies that blinking occurs at time scales larger than the frame acquisition time, i.e. at slower rates in comparison to the frame acquisition rate. As a consequence, one stretch of bright state may be sampled by more than one frames. If blinking occurs at time scales comparable to the frame acquisition time, *μ* ^2^/*σ* ^2^ is less than 0.1 irrespective of the relative lengths of the dark and bright states. Thus, if *M* is not too large, *c*_2_ is comparable to *c*_1_. However, if blinking occurs slower than the frame rate, *μ* ^2^/*σ* ^2^ varies significantly with the relative lengths of the dark and bright states. We include a study in which we investigate *μ* ^2^/*σ* ^2^ as a function of the acquisition time per frame *T* for a given set of ${\tau }_{\max ,b}$ and ${\tau }_{\max ,d}$ in supplementary information S8.

Localization methods explicitly exploit the condition of long dark states (see the curve for ${\tau }_{\max ,b}/{\tau }_{\max ,d}={10}^{-2}$ in Fig. 7(a) for example). The reason is that the ratio *μ* ^2^/*σ* ^2^ is significantly smaller than 1, as seen in Fig. 7(b). Consequently, the image stack is implicitly biased favorably towards the content pertaining super-resolution. In the explicit sense, the emitters remaining in dark state most of the time implies spatial sparsity of emissions in each frame which allows for localization of the emitters. The implicit bias is the reason that long dark states are conducive for non-localization methods as well.

Another extreme condition corresponds to almost no dark states (such as ${\tau }_{\max ,b}/{\tau }_{\max ,d}=100$). This condition is unsuitable for localization microscopy because it implies that the emitters are emitting in most frames and thus spatial sparsity of emitters required in each frame for localization microscopy is unavailable. It is seen in Fig. 7(b) that the *μ* ^2^ and *σ* ^2^ are comparable in such situation if the blinking occurs at larger time scales in comparison to the frame rate.

In most quantum dots, ${\tau }_{\max ,b}$ and ${\tau }_{\max ,d}$ are comparable (see Fig. 7(a) for example ${\tau }_{\max ,b}/{\tau }_{\max ,d}=1$. While such states are undesirable for localization microscopy unless multiple emitter localization techniques such as DAOSTORM^25^ are employed, methods that use fluctuations only can still be conveniently applied to these techniques.

We note that the above example considered the simplified photon emission model of quantum dots whereas diverse effects at multiple time scales participate in the actual photo-kinetics^10, 26^. Fluorescence correlation spectroscopy (FCS) is a useful tool for studying the associated rate constants as well as statistics such as mean (*μ*) and standard deviation (*σ*) of single molecule photo-kinetics^27^. Thus, in practice, single molecule techniques such as FCS can be used to understand and choose situations which yield smaller value of *μ* ^2^/*σ* ^2^ and consequently a larger value of *c*_2_/*c*_1_.

Now, we discuss the role of *M*, which increases linearly with the density of emitters. The value of *c*_1_ increases linearly with *M*, thus scaling up the contribution of the mean image. Here, we note that *M* is the number of emitters in the focal volume, which in context of optical system is approximately the size of one PSF. Also, unlike the convention in localization microscopy, where the emitter density is defined as emitters per unit surface (or volume) per frame, here the density of emitters is the number of emitters per unit surface or volume irrespective of their blinking dynamics. Consider the benchmark examples, MT0.N1.LD and MT0.N1.HD, from the single molecule localization microscopy symposium challenge 2016^28^. The emitter densities per frame for these examples are 0.2 emitters per *μ*m^2^ per frame and 2 emitters per *μ*m^2^ per frame, respectively. However, the value of *M* for these examples is more comparable at 298 and 364 emitters respectively (see supplementary information S9 for details).

## Discussion

Here, we discuss the contemporary super-resolution methods in the framework of the presented eigen-analysis.

### Localization Methods

Localization methods^16–18, 25^, use fluorophores and photo-chemical environment that sustain long dark states and comparatively short bright states (see the curve for ${\tau }_{\max ,b}/{\tau }_{\max ,d}={10}^{-2}$ in Fig. 7(a) for example). It is seen in Fig. 7(b) that *μ* ^2^/*σ* ^2^ is less than 1 for small values of ${\tau }_{\max ,b}/{\tau }_{\max ,d}$. Thus, implicitly the image stack biases the content pertaining super-resolution favorably.

Let the localized emitters be denoted as ${r}_{\tilde{m}}^{^{\prime} }$, $\tilde{m}=1$ to $\tilde{M}$. Let the correspondences be derived between ${r}_{m}^{^{\prime} }$ and ${r}_{\tilde{m}}^{^{\prime} }$ based on the distance between them. Let $\tilde{{\bf{G}}}$ be the analogue of **G** formed using ${r}_{\tilde{m}}^{^{\prime} }$ instead of ${r}_{m}^{^{\prime} }$. Similarly, let ${\bar{G}}_{\tilde{m}}$ be the analogue of ${\bar{G}}_{m}$ and let $\bar{\tilde{\tilde{G}}}$ be the analogue of $\bar{\tilde{G}}$. Further, let ${\tilde{{\bf{C}}}}_{1}=\mathrm{1/(}M\tilde{M})\bar{\tilde{\tilde{G}}}\bar{\tilde{G}}$, ${\tilde{{\bf{C}}}}_{2}=\mathrm{1/}\mathop{M}\limits^{\check{}}\sum _{m=1}^{\mathop{M}\limits^{\check{}}}\,{\bar{G}}_{m}^{{\rm{T}}}{\bar{G}}_{\tilde{m}=m}$, ${\tilde{{\bf{C}}}}_{3}={(\mathop{M}\limits^{\check{}}(\mathop{M}\limits^{\check{}}-1))}^{-1}\sum _{m=1}^{\mathop{M}\limits^{\check{}}}\,\sum _{\tilde{m}\ne m}^{\mathop{M}\limits^{\check{}}}\,{\bar{G}}_{m}^{{\rm{T}}}{\bar{G}}_{\tilde{m}}$, where $\mathop{M}\limits^{\check{}}=\,{\rm{\min }}(M,\tilde{M})$. Considering the least squares methods for localization^29^, the correct localizations for a given noise minimize the distance between the diagonal elements of **C**_2_ and ${\tilde{{\bf{C}}}}_{2}$. Similarly, maximum likelihood estimation (MLE)^30, 31^, primarily uses ${\tilde{{\bf{C}}}}_{3}$ in addition to other non-linear terms consisting of powers of *G*(*n*, *m*) (see supplementary information S10).

We now discuss 3B^15^, which does not impose the restriction of spatio-temporal sparsity strictly. MLE provides an initial localization in 3B. In the absence of sufficient spatio-temporal sparsity, MLE in the initial stage estimates fewer emitters than actually present. From supplementary information S10, it implies that the component ${\tilde{{\bf{C}}}}_{3}$ is not close to **C**_3_ and contributes to poorer maximum value (local maximum) of the log likelihood term maximized in MLE. 3B then simulates blinking as a random Markov process for the previous localizations. The simulation of blinking helps in off-setting this issue as described here. Although one localization may be estimated using MLE instead of few actual emitters, the simulation of temporal blinking statistics of the estimated emitter does not match the blinking statistics of the actual emitters. This indicates the need of localizing more emitters than used in the previous MLE estimation. Thus, in subsequent iterations ${\tilde{{\bf{C}}}}_{3}$ becomes closer to **C**_3_ if the new localizations are more accurate.

### Methods based on statistical moments

Super-resolution optical fluctuation imaging (SOFI) of 2nd order computes^11^:

13 $$F({r}_{n},\kappa )=\mathrm{(1/}K)\sum _{k}\,(I(n,k+\kappa )-{\langle I(n,k)\rangle }_{k})(I(n,k)-{\langle I(n,k)\rangle }_{k})$$

Typically, time lag *κ* is set to zero. In this case, the SOFI image of order 2 is given as the diagonal elements of the matrix ${\bf{F}}={c}_{2}({{\bf{C}}}_{1}+{{\bf{C}}}_{2}-{{\bf{C}}}_{3})$ (derivation in supplementary information S11). SOFI reduces the contribution of **C**_1_ from *c*_1_ to *c*_2_ and thus increasing the weight of super-resolution supporting matrices **C**_2_ and **C**_3_. However, it does not use cross-pixel terms since it uses diagonal components only. Cross-correlated second-order SOFI^12^ and balanced SOFI^13^ incorporate cross-pixel correlations and thus improves the resolution.

ESI^14^ as well as higher order cumulants used in all versions of SOFI^11–13^, include other non-linear terms which appear as powers of *G*(*n*, *m*). Nevertheless, the primary term in ESI and higher order SOFI is common with SOFI of second order and zero time lag. The error of combination of central moments and cumulant is limited by the cumulant of the lowest order^13^. Thus, the roles of **C**_1_, **C**_2_, and **C**_3_ remain the same as second order SOFI.

Balanced SOFI^13^ additionally estimates average emitter blinking characteristics (related to *S*(*m*, *k*), ${\tau }_{\max ,{b}}/({\tau }_{\max ,{d}}+{\tau }_{\max ,{b}})$, and *M*) using non-linear functions of multiple orders of cumulants and applies deconvolution on cumulants to form the super-resolved image. Fourier SOFI^12^ applies a non-linear reweighing function to the Fourier transform of cumulants. Discussion on the effect of non-linear operations is out of the scope of this paper.

### Methods that use eigen-decomposition directly

MUSICAL^21^ computes the eigenvectors of **J** (referred to as eigenimages in ref. 21) and partitions the eigen-space into signal subspace ${\mathcal{S}}$ and null subspace ${\mathcal{N}}$ based whether the singular value *s*_*j*_ (which is the square root of eigenvalue) corresponding to an eigenvector ${\bar{u}}_{j}$ is above a threshold *s*_0_ or not, respectively. The spatial frequencies of the eigenvectors of **J** are the same as the eigenvectors of the circulant matrix **O** since the eigen-decomposition of **O** yields a Fourier transform operator^23^. Further, the spatial frequencies of the eigenvectors increase as the singular value decreases. The eigenvector with the largest eigenvalue is $\bar{\tilde{G}}$. It is the only eigenvector of **C**_1_ and the leading eigenvector of **C**_2_ and **C**_3_. Irrespective of *s*_0_, it is relegated to ${\mathcal{S}}$. Thresholding relegates some of the higher spatial frequency components to ${\mathcal{N}}$. In the presence of noise and large density of emitters (condition *M* > *N* in ref. 21), such relegation is almost inevitable. However, if *M* < *N* and noise power is small, *s*_0_ can be selected such that no eigenvector of **C**_2_ and **C**_3_ is relegated to ${\mathcal{N}}$.

MUSICAL computes $f(r^{\prime} )={({d}_{{\rm{PS}}}(r^{\prime} )/{d}_{{\rm{PN}}}(r^{\prime} ))}^{\alpha }$, where *r*′ is a test point, ${d}_{{\rm{PS}}}(r^{\prime} )={\sum }_{{s}_{j}\ge {s}_{0}}\,\Vert {u}_{j}\cdot \bar{G}(r^{\prime} )\Vert$ is the projection of $\bar{G}(r^{\prime} )$ on ${\mathcal{S}}$, ${d}_{{\rm{PN}}}(r^{\prime} )={\sum }_{{s}_{j} < {s}_{0}}\,\Vert {u}_{j}\cdot \bar{G}(r^{\prime} )\Vert$ is the projection of $\bar{G}(r^{\prime} )$ on ${\mathcal{N}}$, *α* is a constant typically more than 2, $\bar{G}(r^{\prime} )$ is image of a hypothetical emitter at *r*′, and ${d}_{{\rm{PS}}}^{{\rm{2}}}(r^{\prime} )+{d}_{{\rm{PN}}}^{{\rm{2}}}(r^{\prime} )=\Vert \bar{G}(r^{\prime} )\Vert$ and ||·|| represents Euclidean norm. If an emitter is actually present at *r*′, ${d}_{{\rm{PS}}}(r^{\prime} )$ is close to $\Vert \bar{G}(r^{\prime} )\Vert$ and ${d}_{{\rm{PN}}}(r^{\prime} )$ is close to zero. We note that ${d}_{{\rm{PN}}}(r^{\prime} )$ may be non-zero due to high frequency eigenvectors of **C**_2_ and **C**_3_ relegated to ${\mathcal{N}}$. If no emitter is present at *r*′, but *r*′ is close to emitter(s), spatial frequencies of $\bar{G}(r^{\prime} )$ overlap with the spatial frequencies of the eigenvectors in ${\mathcal{S}}$. In other words, the image of such hypothetical emitter overlaps partially with at least one actual emitter. Thus, it results in neither ${d}_{{\rm{PS}}}(r^{\prime} )$ nor ${d}_{{\rm{PN}}}(r^{\prime} )$ close to zero. Lastly, if *r*′ is optically isolated from all emitters such that the spatial frequencies of images of actual emitters hardly overlap with those of the hypothetical emitter at *r*′, ${d}_{{\rm{PS}}}(r^{\prime} )$ is close to zero. The non-linearity introduced due to ${d}_{{\rm{PN}}}(r^{\prime} )$ in denominator and *α* further increases the bias, but the essential exploitation of **C**_2_ and **C**_3_ occurs through the separation of the spatial frequencies in ${\mathcal{S}}$ and ${\mathcal{N}}$.

SCORE^19^, unlike MUSICAL uses only ${\mathcal{S}}$. Thus, while it exploits the spatial frequencies of **C**_2_ and **C**_3_ present in ${\mathcal{S}}$, it rejects the spatial frequencies in ${\mathcal{N}}$. In the case of high emitter density, the spatial frequencies of **C**_2_ and **C**_3_ relegated to the null space have significant role in resolution as discussed above for MUSICAL.

### Concluding remarks

Through the eigen-analysis and the discussion, we establish the roles of emitter distribution, emitter density, and blinking characteristics of emitters in supporting super-resolution. We also identify the component matrices that are exploited by the existing super-resolution algorithms. The presented linear analysis framework can be used to identify the core conceptual drivers of super-resolution and their limiting conditions in the super-resolution techniques that employ blinking. A comprehensive framework that can accommodate non-linear components may be designed in the future. Nevertheless, we expect that new experimental and computational methods may directly exploit these cross-pixel cross-emitter components and push the envelope of super-resolution further.

### Data Availability

The codes used for generating the data in this manuscript are available at https://sites.google.com/site/uthkrishth/musical after the acceptance of the manuscript.

## Electronic supplementary material

Supplementary Information
