# Supplementary material for: Eigen-analysis reveals components supporting super-resolution imaging of blinking fluorophores
Source: Sci Rep. 2017 Jun 30;7:4445. doi: 10.1038/s41598-017-04544-5 (PMC5493635; doi:10.1038/s41598-017-04544-5)
Supplement: Supplementary file 1 — Supplementary Information [file 41598_2017_4544_MOESM1_ESM.pdf]

Eigen-analysis reveals components supporting super-resolution imaging of blinking fluorophores - Supplementary information

Krishna Agarwal[1,2,*] **and Dilip K. Prasad**[3]

[1]BioSystems and Micromechanics Inter-Disciplinary Research Group, Singapore-MIT Alliance for Research and Technology, Singapore 138602
[2]Department of Physics and Technology, UiT-The Arctic University of Norway, 9037 Tromsø, Norway
[3]School of Computer Science and Engineering, Nanyang Technological University, Singapore 639798
[*]uthkrishth@gmail.com

ABSTRACT

See the main paper.

Table Supplementary Table S1. Table of notations

Notation	Description
	Measurements
n, k	pixel number, frame number, respectively
N, K	number of pixels, number of frames, respectively
r, r_n	point in the image space, center of nth pixel, respectively
T	frame acquisition time
$I(n)$, $I(n,k)$	intensity at the nth pixel, intensity at the nth pixel in the kth frame, respectively
$\mathbf{I}$	Recorded image stack (presumably without noise), elements given by $I(n,k)$
	Sample
m, M	emitter number, number of emitters in the focal volume, respectively
r', r'_m	point in the sample space, locations of emitters, respectively
$\tau_{\mathrm{max,b}}$	maximum time scales of the bright states
$\tau_{\mathrm{max,d}}$	maximum time scales of the dark states
$S(m)$, $S(m,k)$	number of photons emitted by the mth emitter, number of photons emitted by the mth emitter during the kth frame, respectively
$\bar{S}_m$	blinking vector of mth emitter, containing $S(m,k)$
$\mathbf{S}$	blinking matrix containing photon emissions from all emitters in each frame, elements given by $S(m,k)$
μ,σ	mean and standard deviation of the photon counting statistics of an emitter, respectively
	Imaging system
$g(r,r')$	mapping function that maps the emission of a photon at r' to the intensity at r
$G(n,m)$	emitter-to-pixel mapping, given in eq. (S.2)
$\bar{G}_m$	image of the mth emitter, contains $G(n,m)$
$\bar{G}(r')$	image of an emitter at r', contains $G(r_n,r')$
$\mathbf{G}$	mapping matrix, contains mapping vectors of all emitters, elements given by $G(n,m)$

S1 Imaging model

Let there be a distribution of M stationary emitters in the focal volume of the sample space. The points in the sample space are denoted by r' and the location of the mth emitter is denoted by r'_m. The points in the image plane are denoted by r, the

pixel centers are denoted by r_n, and the region of one pixel is denoted by Ω_n. Let the PSF of the imaging system be denoted as $g(r, r')$. $g(r, r')$ maps the emission at a point r' in the sample plane to the intensity at a point r in the sample plane.

The intensity at the nth pixel due to all emitters is given as:

$$I(n) = \int_{r\in\Omega_n} \sum_{m=1}^{M} g(r, r'_m) S(m) \mathrm{d}r = \sum_{m=1}^{M} S(m) \int_{r\in\Omega_n} g(r, r'_m) \mathrm{d}r \tag{S.1}$$

where $S(m)$ denotes the number of photons emitted by the mth emitter during one image acquisition. We define an emitter-to-pixel mapping $G(n,m)$ as follows:

$$G(n,m) = \int_{r\in\Omega_n} g(r, r'_m) \mathrm{d}r \tag{S.2}$$

Thus, eq. (S.1) can be simplified as:

$$I(n) = \sum_{m=1}^{M} G(n,m) S(m) \tag{S.3}$$

The intensities $I(n)$ at all the pixels, usually arranged in a 2-dimensional array, comprise an image. For this analysis, the intensities at all the pixels in an image are collected into a column vector such that there is one column vector for each frame. Thus, the complete image stack is represented as a two-dimensional matrix $\mathbf{I}$ in which row n corresponds to the nth pixel, column k corresponds to the kth frame, and element $I(n,k)$ corresponds to intensity at the nth pixel in the kth frame. The matrix $\mathbf{I}$ has dimension $N \times K$, where N is the total number of pixels and K is the total number of frames. $\mathbf{I}$ can be represented in matrix form as follows:

$$\mathbf{I} = \mathbf{GS} \tag{S.4}$$

where $\mathbf{G}$ is a matrix of dimension $N \times M$ in which the (n,m)th element is $G(n,m)$ and $\mathbf{S}$ is a matrix of dimension $M \times K$ in which the (m,k)th element is $S(m,k)$, i.e. number of photons emitted by the mth emitter in the kth frame.

For later convenience, we denote the matrices $\mathbf{G}$ and $\mathbf{S}$ as:

$$\mathbf{G} = \begin{bmatrix} \bar{G}_1 & \bar{G}_2 & \cdots & \bar{G}_M \end{bmatrix} \tag{S.5}$$

$$\mathbf{S} = \begin{bmatrix} \bar{S}_1 & \bar{S}_2 & \cdots & \bar{S}_M \end{bmatrix}^{\mathrm{T}} \tag{S.6}$$

where $\bar{G}_m$ is the image of the mth emitter. Similarly, $\bar{S}_m$ is blinking vector for the mth emitter. We also define:

$$\bar{\bar{G}} = \sum_m \bar{G}_m = \mathbf{G}\mathscr{L}_{M\times 1} \tag{S.7}$$

The mean image of the image stack is given as:

$$\bar{\bar{I}} = \frac{1}{K}\mathbf{I}\mathscr{L}_{K\times 1} \tag{S.8}$$

Substituting eq. S.4 in the above, we get:

$$\bar{\bar{I}} = \frac{1}{K}\mathbf{GS}\mathscr{L}_{K\times 1} \tag{S.9}$$

$\mathbf{S}\mathscr{L}_{K\times 1}$ contains $K\langle S(m,k)\rangle_k$. Since all the emitters are similar according to assumption A1, $\langle S(m,k)\rangle_k = \mu \forall m$. Thus,

$$\bar{\bar{I}} = \mu\mathbf{G}\mathscr{L}_{M\times 1} \tag{S.10}$$

Using eq. (S.7),

$$\bar{\bar{I}} = \mu\bar{\bar{G}} \tag{S.11}$$

S2 Derivation from eq. (1) to eq. (2)

Substituting eq. (S.4) in eq. (1), we get:

$$\mathbf{J} = \mathbf{G}\mathbf{S}\mathbf{S}^{\mathrm{T}}\mathbf{G}^{\mathrm{T}} \tag{S.12}$$

$$= \mathbf{G}\begin{bmatrix} \bar{S}_1^{\mathrm{T}}\bar{S}_1 & \bar{S}_1^{\mathrm{T}}\bar{S}_2 & \cdots & \bar{S}_1^{\mathrm{T}}\bar{S}_M \\ \bar{S}_2^{\mathrm{T}}\bar{S}_1 & \ddots & & \\ \vdots & & \ddots & \\ \bar{S}_M^{\mathrm{T}}\bar{S}_1 & & & \bar{S}_M^{\mathrm{T}}\bar{S}_M \end{bmatrix}\mathbf{G}^{\mathrm{T}} \tag{S.13}$$

$$= K\mathbf{G}\begin{bmatrix} \langle S(1,k)S(1,k)\rangle_k & \langle S(1,k)S(2,k)\rangle_k & \cdots & \langle S(1,k)S(M,k)\rangle_k \\ \langle S(2,k)S(1,k)\rangle_k & \ddots & & \\ \vdots & & \ddots & \\ \langle S(M,k)S(1,k)\rangle_k & & & \langle S(M,k)S(M,k)\rangle_k \end{bmatrix}\mathbf{G}^{\mathrm{T}} \tag{S.14}$$

Let x and y be two random variables. Their means are $\langle x\rangle$ and $\langle y\rangle$, respectively. The mean-shifted variables are $\dot{x} = x - \langle x\rangle$ and $\dot{y} = y - \langle y\rangle$. Obviously, $\langle \dot{x}\rangle = 0$ and $\langle \dot{y}\rangle = 0$. Then, $\langle xy\rangle = \langle \dot{x}\dot{y}\rangle + \langle x\rangle\langle y\rangle$. Covariance is $\langle \dot{x}\dot{y}\rangle$. Thus, $\langle S(m_1,k)S(m_2,k)\rangle_k = \langle \dot{S}(m_1,k)\dot{S}(m_2,k)\rangle_k + \langle S(m_1,k)\rangle_k\langle S(m_2,k)\rangle_k$. Since all emitters are similar according to assumption A1 and the number of frames is sufficiently large according to A3, $\langle S(m_1,k)\rangle_k = \langle S(m_2,k)\rangle_k = \mu$. Thus, $\mathbf{J}$ can be simplified as:

$$= K\mathbf{G}\left(\mathbf{R} + \mu^2\mathscr{L}_{M\times M}\right)\mathbf{G}^{\mathrm{T}} \tag{S.15}$$

where $\mathbf{R}$ is the covariance matrix. The diagonal elements of $\mathbf{R}$ are standard deviations $\langle \dot{S}(m,k)\dot{S}(m,k)\rangle_k = \sigma^2$, where assumption A3 is used. Further, since blinking of one emitter is independent of any other emitter according to assumption A2, covariance $\langle \dot{S}(m_1,k)\dot{S}(m_2,k)\rangle_k = 0$ for $m_1 \neq m_2$. Thus, $\mathbf{R} = \sigma^2\mathscr{I}_M$. Thus, eq. (S.15) can be written as:

$$\mathbf{J} = K\mathbf{G}\left(\sigma^2\mathscr{I}_M + \mu^2\mathscr{L}_{M\times M}\right)\mathbf{G}^{\mathrm{T}} \tag{S.16}$$

S3 Eigen-analysis of circulant matrix O

Matrix $\mathbf{O}$ is a circulant matrix of size M and has the form:

$$O(m',m) = \begin{matrix} a_1 & \text{if } m = m' \\ a_0 & \text{if } m \neq m' \end{matrix} \tag{S.17}$$

where

$$a_0 = \mu^2; \qquad a_1 = \sigma^2 + \mu^2 \tag{S.18}$$

The eigenvalues α_j and eigenvectors $\bar{v}_j$ of such circulant matrix are computed as [S1]:

$$\alpha_j = a_1 + a_0\left(\omega_j + \omega_j^2 + \cdots + \omega_j^{M-1}\right) \tag{S.19}$$

$$\bar{v}_j = \frac{1}{\sqrt{M}}\begin{bmatrix} 1 & \omega_j & \omega_j^2 & \cdots & \omega_j^{M-1} \end{bmatrix}^{\mathrm{T}}; \tag{S.20}$$

where $i = \sqrt{-1}$ and

$$\omega_j = \exp\left(i\frac{2\pi(j-1)}{M}\right) \tag{S.21}$$

Further, since ω_j is Mth root of unity, it satisfies:

$$\left(\omega_j + \omega_j^2 + \cdots + \omega_j^{M-1}\right) = \begin{matrix} M-1 & \text{if } j = 1 \\ -1 & \text{otherwise} \end{matrix} \tag{S.22}$$

Consequently, $\alpha_1 = a_1 + (M-1)a_0 = \sigma^2 + M\mu^2$ and $\alpha_{j>1} = a_1 - a_0 = \sigma^2$. We assign $\alpha_0 = a_1 - a_0$.

As a consequence of the eigen-decomposition, $\mathbf{O}$ can be written as:

$$\mathbf{O} = \mathbf{V}^{\dagger}\mathbf{A}\mathbf{V} \tag{S.23}$$

where, the superscript $\dagger$ is the Hermitian operator, $\mathbf{A} = \operatorname{diag}\left([\alpha_1 \quad \alpha_0 \quad \ldots \quad \alpha_0]\right)$ and

$$\mathbf{V} = \frac{1}{\sqrt{M}}\begin{bmatrix} \bar{v}_1 & \bar{v}_2 & \cdots & \bar{v}_M \end{bmatrix} = \begin{bmatrix} 1 & 1 & 1 & 1 & \cdots & 1 \\ 1 & \varpi & \varpi^2 & \varpi^3 & \cdots & \varpi^{(M-1)} \\ 1 & \varpi^2 & \varpi^4 & \varpi^6 & \cdots & \varpi^{2(M-1)} \\ 1 & \varpi^3 & \varpi^6 & \varpi^9 & \cdots & \varpi^{3(M-1)} \\ \vdots & \vdots & \vdots & \vdots & \ddots & \vdots \\ 1 & \varpi^{(M-1)} & \varpi^{2(M-1)} & \varpi^{3(M-1)} & \cdots & \varpi^{(M-1)^2} \end{bmatrix} \tag{S.24}$$

where $\varpi = \exp(2\pi i/M)$. We note that the matrix $\mathbf{V}$ is related to discrete Fourier transform operation [S1]. Eq. (S.24) shows that $\mathbf{V}$ is symmetric, i.e. $\mathbf{V}^{\mathrm{T}} = \mathbf{V}$. Thus, $\mathbf{V}^{\dagger} = \operatorname{conj}\left(\mathbf{V}^{\mathrm{T}}\right) = \operatorname{conj}(\mathbf{V})$. We define $\mathbf{U} = \begin{bmatrix} \bar{u}_1 & \bar{u}_2 & \cdots & \bar{u}_M \end{bmatrix}$ where $\bar{u}_j = \operatorname{conj}(\bar{v}_j)$, such that $\mathbf{V}^{\dagger} = \mathbf{U}$ and $\mathbf{O} = \mathbf{U}\mathbf{A}\mathbf{V}$. Further, using the symmetry of $\mathbf{V}$, we write $\mathbf{O} = \mathbf{U}\mathbf{A}\mathbf{V}^{\mathrm{T}}$. Subsequently,

$$\mathbf{O} = \begin{bmatrix} \bar{u}_1 & \bar{u}_2 & \cdots & \bar{u}_M \end{bmatrix} \operatorname{diag}\left([\alpha_1 \quad \alpha_0 \quad \ldots \quad \alpha_0]\right) \begin{bmatrix} \bar{v}_1^{\mathrm{T}} \\ \bar{v}_2^{\mathrm{T}} \\ \vdots \\ \bar{v}_M^{\mathrm{T}} \end{bmatrix} = \alpha_1 \bar{u}_1 \bar{v}_1^{\mathrm{T}} + \alpha_0 \sum_{j=2}^{M} \bar{u}_j \bar{v}_j^{\mathrm{T}} \tag{S.25}$$

S4 Derivation from eq. (2) to eq. (4)

Here, we use the results of S3 implicitly. Using eq. (S.24), the elements of eigenvector $\bar{v}_j$ of $\mathbf{O}$ are given by

$$v_{j,j'} = a_{j,j'}/\sqrt{M} \tag{S.26}$$

where

$$a_{j,j'} = \varpi^{(j-1)(j'-1)} = \exp\left(\frac{2\pi i}{M}(j-1)(j'-1)\right) \tag{S.27}$$

We also note that $\bar{u}_1 = \bar{v}_1 = M^{-1/2}\mathscr{L}_{M\times 1}$. Substituting eq. (S.25) in eq. (2), we get:

$$\mathbf{J} = K\alpha_1(\mathbf{G}\bar{u}_1)(\mathbf{G}\bar{v}_1)^{\mathrm{T}} + K\alpha_0\sum_{j=2}^{M}(\mathbf{G}\bar{u}_j)(\mathbf{G}\bar{v}_j)^{\mathrm{T}} \tag{S.28}$$

Further, we compute $\mathbf{G}\bar{v}_j$ and $\mathbf{G}\bar{u}_j$ as follows:

$$\mathbf{G}\bar{v}_j = \frac{1}{\sqrt{M}}\sum_{m=1}^{M}\bar{G}_m a_{j,m} = \frac{1}{\sqrt{M}}\sum_{m=1}^{M}\bar{G}_m \exp\left(\frac{2\pi i}{M}(j-1)(m-1)\right) \tag{S.29}$$

$$\mathbf{G}\bar{u}_j = \mathbf{G}\operatorname{conj}(\bar{v}_j) = \frac{1}{\sqrt{M}}\sum_{m'=1}^{M}\bar{G}_{m'}\operatorname{conj}(a_{j,m'}) = \frac{1}{\sqrt{M}}\sum_{m'=1}^{M}\bar{G}_{m'}\exp\left(-\frac{2\pi i}{M}(j-1)(m'-1)\right) \tag{S.30}$$

Consequently,

$$(\mathbf{G}\bar{u}_j)(\mathbf{G}\bar{v}_j)^{\mathrm{T}} = \frac{1}{M}\sum_{m=1}^{M}\sum_{m'=1}^{M}\bar{G}_{m'}\bar{G}_m^{\mathrm{T}}\exp\left(\frac{2\pi i}{M}(j-1)(m-m')\right) \tag{S.31}$$

and eq. (S.28) can be written as:

$$\mathbf{J} = \frac{K}{M}\alpha_1\bar{\bar{G}}\bar{\bar{G}}^{\mathrm{T}} + \frac{K}{M}\alpha_0\sum_{m=1}^{M}\sum_{m'=1}^{M}\bar{G}_{m'}\bar{G}_m^{\mathrm{T}}\sum_{j=2}^{M}\exp\left(\frac{2\pi i}{M}(j-1)(m-m')\right) \tag{S.32}$$

Here, we have used eq. (S.7) for the first term. We simplify the second summation term by assigning:

$$C(m,m') = \sum_{j=2}^{M} \exp\left(i\theta(j-1)\right); \quad \theta = \frac{2\pi}{M}(m-m') \tag{S.33}$$

and solving it for the cases $m = m'$ and $m \neq m'$ separately. For $m = m'$, $C(m,m') = (M-1)$ since $\theta = 0$.

For $m \neq m'$, $C(m,m' \neq m)$ can be interpret as a geometric series with common ratio r and initial value a given by $r = a = \exp(i\theta)$ and number of elements in series equal to $M-1$. Thus, $C(m,m' \neq m)$ can be written as

$$C(m,m' \neq m) = a\frac{1-r^{M-1}}{1-r} = \exp(i\theta)\,\frac{1-\exp\left(i\theta(M-1)\right)}{1-\exp(i\theta)} \tag{S.34}$$

Using algebra of complex numbers, the above can be simplified to

$$C(m,m' \neq m) = \exp\left(i\theta\frac{M}{2}\right)\frac{\sin\frac{(M-1)\theta}{2}}{\sin\frac{\theta}{2}} \tag{S.35}$$

It is notable that $\theta M/2 = \pi(m-m')$ and $(m-m')$ being an integer, we get $\exp(i\theta M/2) = (-1)^{(m-m')}$. Further, using $\sin(M-1)\theta/2 = \cos\theta/2\sin M\theta/2 - \sin\theta/2\cos M\theta/2$, $\sin M\theta/2 = 0$, and $\cos M\theta/2 = (-1)^{(m-m')}$, we simplify eq. (S.35) as $C(m,m' \neq m) = -1(-1)^{2(m-m')} = -1$.

Thus, substituting the values of $C(m,m' = m)$ and $C(m,m' \neq m)$ in eq. (S.32), we get:

$$\mathbf{J} = \frac{K}{M}\alpha_1\bar{\tilde{G}}\bar{\tilde{G}}^{\mathrm{T}} + \frac{K}{M}(M-1)\alpha_0\sum_{m=1}^{M}\bar{G}_m\bar{G}_m^{\mathrm{T}} - \frac{K}{M}\alpha_0\sum_{m=1}^{M}\sum_{m'=1,\neq m}^{M}\bar{G}_{m'}\bar{G}_m^{\mathrm{T}} \tag{S.36}$$

S5 Relaxation of assumption A2

The assumption A2 is first used after eq. (S.15). If the assumption A2 is relaxed, i.e. blinking of emitters need not be independent of other emitters, then **R** is not a diagonal matrix. It is a circulant matrix with diagonal elements equal to σ^2 (the variance of photon emission distribution of an emitter) and off-diagonal elements of $\tilde{\sigma}^2$ (the covariance of photon emission distributions of any two emitter). Consequently, with reference to S3, **O** is a circulant matrix with diagonal elements equal to $a_1 = \sigma^2 + \mu^2$ and off-diagonal elements equal to $a_0 = \tilde{\sigma}^2 + \mu^2$. The derivations in S3 apply directly, with the revised values of $\alpha_1 = (\sigma^2 - \tilde{\sigma}^2) + M(\mu^2 + \tilde{\sigma}^2)$ and $\alpha_0 = (\sigma^2 - \tilde{\sigma}^2)$. Then, following the analysis in S4, eq. (5) can be written as:

$$c_1 = KM\left((\sigma^2 - \tilde{\sigma}^2) + M(\mu^2 + \tilde{\sigma}^2)\right); \quad c_2 = K(M-1)(\sigma^2 - \tilde{\sigma}^2) \tag{S.37}$$

S6 Simulation of examples illustrating $\mathbf{C}_{\{1,2,3\}}$

In this example, two emitters are along the $y-$axis in the lateral plane such that $y'_1 = -\Delta y/2$ and $y'_2 = \Delta y/2$ are their locations. The emission wavelength is 510 nm (which corresponds to the green fluorescence proteins), the numerical aperture is 1.4, and the PSF is given by the Airy function [S2]. The PSF is normalized by its maximum amplitude before computing all the matrices.

S7 Model and simulation of photon counting statistics

It was shown in [S3] that the photon-count statistics due to blinking can be modelled as sum of two Poisson distributions and a third largely-flat low probability term. The two Poisson distributions correspond to the emissions in dark states and the emission in bright states respectively. They have means equal to $n_{\mathrm{d}}T$ and $n_{\mathrm{b}}T$ where T is the time per frame, and n_{d} and n_{b} are the fluorescence emission rates of the dark and bright state, respectively. The third term corresponds to the jumps in the state of molecule during the acquisition of one frame. For very slow acquisition, this third term converges to a Gaussian distribution. However, this is seldom the case in super-resolution imaging and the two Poisson distributions dominate the photon-count statistics [S3]. Thus, the photon counting statistics is modelled as:

$$P(s|T) \approx p_{\mathrm{d}}\mathrm{Poi}(n_{\mathrm{d}}T) + p_{\mathrm{b}}\mathrm{Poi}(n_{\mathrm{b}}T) \tag{S.38}$$

where $\text{Poi}(X)$ denotes Poisson distribution in random variable s with mean X, p_d and p_b are the mixing proportions for the Poisson distributions corresponding to the dark and bright states. The proportions p_d and p_b are functions of the quantum yield y, the smallest and largest time scales of the dark states, $\tau_{\text{min,d}}$ and $\tau_{\text{max,d}}$ respectively, the smallest and largest time scales of the bright states, $\tau_{\text{min,b}}$ and $\tau_{\text{max,b}}$ respectively, and the power terms α_d and α_b of the power laws governing the statistics of the times of dark and bright states. The proportion p_d is given as:

$$\begin{aligned} p_\text{d} &= cN_\text{b} \sum_{j=1}^{j=N_\text{d}} \frac{exp(-K_{j,\text{d}}T)}{K_{j,\text{d}}} \\ p_\text{b} &= cN_\text{d} \sum_{j=1}^{j=N_\text{b}} \frac{exp(-K_{j,\text{b}}T)}{K_{j,\text{b}}} \end{aligned} \quad (S.39)$$

where

$$\begin{aligned} K_{j,\text{d}} &= \left(K_{1,\text{d}}^{\alpha_d-1} + A_\text{d}(j-1)\right)^{\frac{1}{\alpha_d}}; \quad A_\text{d} = \frac{K_{N_\text{d},\text{d}}^{\alpha_\text{d}-1} - K_{1,\text{d}}^{\alpha_\text{d}-1}}{N_\text{d}-1}; \quad K_{1,\text{d}} = 1/\tau_{\text{max,d}}; \quad K_{N_\text{d},\text{d}} = 1/\tau_{\text{min,d}}; \\ K_{j,\text{b}} &= \left(K_{1,\text{b}}^{\alpha_b-1} + A_\text{b}(j-1)\right)^{\frac{1}{\alpha_b}}; \quad A_\text{b} = \frac{K_{N_\text{b},\text{b}}^{\alpha_\text{b}-1} - K_{1,\text{b}}^{\alpha_\text{b}-1}}{N_\text{b}-1}; \quad K_{1,\text{b}} = 1/\tau_{\text{max,b}}; \quad K_{N_\text{b},\text{b}} = 1/\tau_{\text{min,b}}; \end{aligned} \quad (S.40)$$

$$c = \left(N_b \sum_{j=1}^{j=N_\text{d}} \frac{1}{K_{j,\text{d}}} + N_d \sum_{j=1}^{j=N_\text{b}} \frac{1}{K_{j,\text{b}}}\right)^{-1} \quad (S.41)$$

$$N_\text{b} = yN_\text{d} \quad (S.42)$$

and N_d is sufficiently large. We found that the ratios μ^2/σ^2 are not sensitive to the value of y. We have used $y = 0.5$, $T = 10$ ms, $n_d = 1$ photons/ms, $n_b = 100$ photons/ms, $\alpha_\text{d} = \alpha_\text{b} = 1.5$, $N_\text{d} = 10^6$, and $\tau_{\text{min,d}} = \tau_{\text{min,b}} = 0.01$ ms.

S8 μ^2/σ^2 as a function of acquisition time per frame T

Here, we study μ^2/σ^2 as a function of the acquisition time per frame T for a several sets of $\tau_{\text{max,b}}$ and $\tau_{\text{max,d}}$ shown in Supplementary Figure S1. We consider the values of T in the range $[\tau_{\text{max,b}}, \tau_{\text{max,d}}]$. $T < \tau_{\text{max,b}}$ corresponds to the case where the measured data is expected to be temporally sparse while $T > \tau_{\text{max,d}}$ corresponds to the case where the measured data is expected to demonstrate rapidly decreasing temporal sparsity. In Supplementary Figure S1, comparison of the plots in dashed lines indicates that pattern is repeatable for $\tau_{\text{max,b}}/\tau_{\text{max,d}} = 0.01$, which correspond to long dark states. Similar inference applies to the solid lines that correspond to $\tau_{\text{max,b}}/\tau_{\text{max,d}} = 0.1$, i.e. relatively shorter dark states. Interestingly, the value of μ^2/σ^2 exhibits a maximum for the case $\tau_{\text{max,b}}/\tau_{\text{max,d}} = 0.01$ and a minimum for the case $\tau_{\text{max,b}}/\tau_{\text{max,d}} < 0.1$. This implies that there is a worst value of T for the case $\tau_{\text{max,b}}/\tau_{\text{max,d}} = 0.01$ and a best value of T for the case $\tau_{\text{max,b}}/\tau_{\text{max,d}} = 0.1$. Also, the lowest value of μ^2/σ^2 for the case $\tau_{\text{max,b}}/\tau_{\text{max,d}} = 0.1$ is much lower than the highest value of μ^2/σ^2 for the case $\tau_{\text{max,b}}/\tau_{\text{max,d}} = 0.01$. This provides a counter-intuitive inference that shorter dark states may correspond to a larger value of c_2/c_1 for a suitably chosen T, which consequently indicates better possibility of super-resolution. However, such observations have been reported before as well [S4].

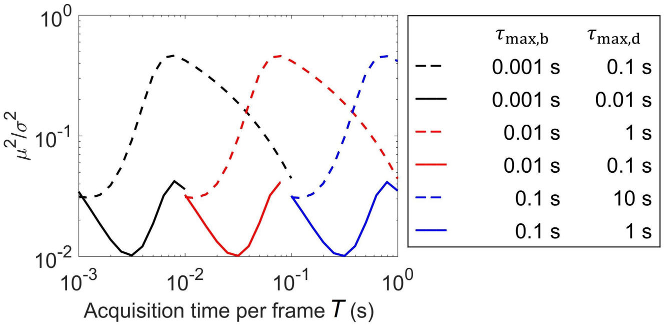

Figure Supplementary Figure S1. μ^2/σ^2 as a function of the acquisition time per frame T for a given set of $\tau_{\text{max,b}}$ and $\tau_{\text{max,d}}$.

S9 Estimation of the value of M for the benchmark examples MT0.N1.LD and MT0.N1.HD in single molecule localization microscopy symposium challenge 2016 [S5]

MT0.N1.LD is an example of simulated 3-dimensional arrangement of microtubules with low density of emitters per frame, specifically 0.2 emitters per μm^3 per frame. It has a total of 8731 emitters in a square field of view (emitters projected on the lateral $x-y$ plane) of $6.4 \times 6.4\ \mu$m^2. However, since most of the region is empty, we consider the median number of emitters when the field of view is discretized into squares of dimensions $\lambda/$NA (approximately the width of the PSF). This number is multiplied by $\pi/4$ to obtain median number of emitters in circular region or radius $\lambda/2$NA. This can be used as an approximate value of M for analysis presented in this paper. The value of M for MT0.N1.LD is 298.

MT0.N1.HD is another benchmark example similar to of MT0.N1.LD, but with high density of emitters per frame, which is 2 emitters per μm^3 per frame. It has a total of 11172 emitters in a square field of view of $6.4 \times 6.4\ \mu$m^2. The value of M for this example computed as described above is 364.

S10 Implication of maximum likelihood estimation of localization

In maximum likelihood estimation, the following log likelihood is maximized [S6, S7].

$$\ln L(\bar{I}_m|\{r'_{\tilde{m}}, S(\tilde{m})\}) = \sum_n (I_m(n)\ln S(\tilde{m})G(n,\tilde{m}) - S(\tilde{m})G(n,\tilde{m}) - I_m(n)!) \tag{S.43}$$

where $\bar{I}_m$ is the measured image of the mth emitter (assuming emissions from only the mth emitter in the focal volume) with elements $I_m(n)$ and $r'_{\tilde{m}}$ and $S(\tilde{m})$ are the estimated location and the estimated number of photons emitted by the mth emitter. Here noise terms are suppressed for simplicity. Analogously, for multiple emitters in an image, the MLE problem is written as:

$$\ln L(\bar{I}|\{r'_{\tilde{m}}, S(\tilde{m}); \forall m\}) = \sum_n \left(I(n)\sum_m \ln S(\tilde{m})G(n,\tilde{m}) - \sum_m S(\tilde{m})G(n,\tilde{m}) - I(n)! \right) \tag{S.44}$$

Using Taylor series expansion for $\ln S(\tilde{m})G(n,\tilde{m})$ in eq. (S.43), we get:

$$\begin{aligned}\ln L(\bar{I}|\{r'_{\tilde{m}}, S(\tilde{m}); \forall m\}) = \quad & \overbrace{\sum_n I(n)\sum_m S(\tilde{m})G(n,\tilde{m})}^{\text{Term1}} + \overbrace{\sum_n\sum_m S(\tilde{m})G(n,\tilde{m}))}^{\text{Term2}} \\ & \overbrace{+\sum_n\sum_m\sum_{j=2}^{\infty}\left(\frac{(-1)^{j-1}}{j} I(n)(S(\tilde{m})G(n,\tilde{m})-1)^j\right)}^{\text{Term3}} \\ & \overbrace{-\sum_n I(n) - \sum_n I(n)!}^{\text{Term4}}\end{aligned} \tag{S.45}$$

Term 4 does not participate in maximization since it is not a function of $\tilde{m}$. Term 3 is a non-linear term, in fact a power series. Term 2 corresponds to the term linear in $G(n,\tilde{m})$ independent of measurements and incomparable with the matrices $\tilde{\mathbf{C}}_1$, $\tilde{\mathbf{C}}_2$, and $\tilde{\mathbf{C}}_3$.

Term 1 is directly associated with the matrices $\tilde{\mathbf{C}}_2$ and $\tilde{\mathbf{C}}_3$ as we show below. The first term $\sum_n I(n)\sum_m s(\tilde{m})G(n,\tilde{m})$ can be given as $\bar{S}^{\mathrm{T}}\mathbf{G}^{\mathrm{T}}\tilde{\mathbf{G}}\bar{\bar{S}}$, where $\bar{S}$ is the vector containing the number of photons emitted by the emitters and $\bar{\bar{S}}$ is an estimate of it. Further, with algebraic manipulations, $\mathbf{G}\tilde{\mathbf{G}} = \min(M,\tilde{M})\tilde{\mathbf{C}}_2 + \min(M,\tilde{M})(\min(M,\tilde{M})-1)\tilde{\mathbf{C}}_3 = \min(M,\tilde{M})(\tilde{\mathbf{C}}_2 - \tilde{\mathbf{C}}_3) + \min(M^2,\tilde{M}^2)\tilde{\mathbf{C}}_3$. Thus, the first term incorporates $\tilde{\mathbf{C}}_2 - \tilde{\mathbf{C}}_3$ and an additional component of $\tilde{\mathbf{C}}_3$. This implies that although the magnitude of $\tilde{\mathbf{C}}_2 - \tilde{\mathbf{C}}_3$ may be small, MLE incorporates the component $\tilde{\mathbf{C}}_3$ which is the richest in numerical content pertaining super-resolution.

S11 Derivation of $\mathbf{F}$ for SOFI

We define:

$$\mathbf{F} = (\mathbf{I} - \bar{\bar{I}}\mathscr{L}^{\mathrm{T}}_{K\times 1})(\mathbf{I} - \bar{\bar{I}}\mathscr{L}^{\mathrm{T}}_{K\times 1})^{\mathrm{T}} \tag{S.46}$$

where $\bar{\bar{I}}$ is the mean image and is given by eqs. (S.8,S.11). Thus, $\mathbf{F}$ can be expanded as

$$\begin{aligned}\mathbf{F} &= \mathbf{J} - \bar{\bar{I}}\mathscr{L}^{\mathrm{T}}_{K\times 1}\mathbf{I}^{\mathrm{T}} - \mathbf{I}\mathscr{L}_{K\times 1}\bar{\bar{I}}^{\mathrm{T}} + \bar{\bar{I}}\mathscr{L}^{\mathrm{T}}_{K\times 1}\mathscr{L}_{K\times 1}\bar{\bar{I}}^{\mathrm{T}} \\ &= \mathbf{J} - 2K\bar{\bar{I}}\bar{\bar{I}}^{\mathrm{T}} + K\bar{\bar{I}}\bar{\bar{I}}^{\mathrm{T}} \\ &= \mathbf{J} - K\mu^2\tilde{\mathbf{G}}\tilde{\mathbf{G}}^{\mathrm{T}} \\ &= \mathbf{J} - K\mu^2M^2\mathbf{C}_1 = c_2(\mathbf{C}_1 + \mathbf{C}_2 - \mathbf{C}_3)\end{aligned} \tag{S.47}$$

where eqs. (4−8) have been used.

References

[S1] Golub, G. H. & Van Loan, C. F. *Matrix Computations*, vol. 3 (JHU Press, 2012).

[S2] Born, M. & Wolf, E. *Principles of optics: electromagnetic theory of propagation, interference and diffraction of light* (Cambridge University Press, 1999).

[S3] Volkán-Kacsó, S. Two-state theory of binned photon statistics for a large class of waiting time distributions and its application to quantum dot blinking. *The Journal of Chemical Physics* **140**, 224110 (2014).

[S4] Lin, Y. *et al.* Quantifying and optimizing single-molecule switching nanoscopy at high speeds. *PloS one* **10**, e0128135 (2015).

[S5] Biomedical Imaging Group, Ecole Polytechnique Fédérale de Lausanne, Switzerland. Single Molecule Localization Microscopy Synposium Challenge 2016. http://bigwww.epfl.ch/smlm/challenge2016/ (2016). [Online; accessed 17-January-2017].

[S6] Ober, R. J., Ram, S. & Ward, E. S. Localization accuracy in single-molecule microscopy. *Biophysical Journal* **86**, 1185–1200 (2004).

[S7] Smith, C. S., Joseph, N., Rieger, B. & Lidke, K. A. Fast, single-molecule localization that achieves theoretically minimum uncertainty. *Nature Methods* **7**, 373–375 (2010).
